# Social robots in advanced dementia

**DOI:** 10.3389/fnagi.2015.00133

**Published:** 2015-09-03

**Authors:** Meritxell Valentí Soler, Luis Agüera-Ortiz, Javier Olazarán Rodríguez, Carolina Mendoza Rebolledo, Almudena Pérez Muñoz, Irene Rodríguez Pérez, Emma Osa Ruiz, Ana Barrios Sánchez, Vanesa Herrero Cano, Laura Carrasco Chillón, Silvia Felipe Ruiz, Jorge López Alvarez, Beatriz León Salas, José M. Cañas Plaza, Francisco Martín Rico, Gonzalo Abella Dago, Pablo Martínez Martín

**Affiliations:** ^1^Research Unit, CIEN Foundation, Carlos III Institute of Health, Alzheimer Center Reina Sofía FoundationMadrid, Spain; ^2^CIBERSAM-Carlos III Institute of Health MadridMadrid, Spain; ^3^Maria Wolff FoundationMadrid, Spain; ^4^Health Care Area, Alzheimer Center Reina Sofía FoundationMadrid, Spain; ^5^RoboticsLab, Rey Juan Carlos UniversityMadrid, Spain; ^6^CIBERNED-Carlos III Institute of HealthMadrid, Spain; ^7^Applied Research Area, National Center of Epidemiology, Carlos III Institute of HealthMadrid, Spain

**Keywords:** dementia, Alzheimer disease, therapy, robotics, human-robot interaction, technology, animal assisted therapy, apathy

## Abstract

**Aims:** Pilot studies applying a humanoid robot (NAO), a pet robot (PARO) and a real animal (DOG) in therapy sessions of patients with dementia in a nursing home and a day care center.

**Methods:**In the nursing home, patients were assigned by living units, based on dementia severity, to one of the three parallel therapeutic arms to compare: CONTROL, PARO and NAO (Phase 1) and CONTROL, PARO, and DOG (Phase 2). In the day care center, all patients received therapy with NAO (Phase 1) and PARO (Phase 2). Therapy sessions were held 2 days per week during 3 months. Evaluation, at baseline and follow-up, was carried out by blind raters using: the Global Deterioration Scale (GDS), the Severe Mini Mental State Examination (sMMSE), the Mini Mental State Examination (MMSE), the Neuropsychiatric Inventory (NPI), the Apathy Scale for Institutionalized Patients with Dementia Nursing Home version (APADEM-NH), the Apathy Inventory (AI) and the Quality of Life Scale (QUALID). Statistical analysis included descriptive statistics and non-parametric tests performed by a blinded investigator.

**Results:** In the nursing home, 101 patients (Phase 1) and 110 patients (Phase 2) were included. There were no significant differences at baseline. The relevant changes at follow-up were: (Phase 1) patients in the robot groups showed an improvement in apathy; patients in NAO group showed a decline in cognition as measured by the MMSE scores, but not the sMMSE; the robot groups showed no significant changes between them; (Phase 2) QUALID scores increased in the PARO group. In the day care center, 20 patients (Phase 1) and 17 patients (Phase 2) were included. The main findings were: (Phase 1) improvement in the NPI irritability and the NPI total score; (Phase 2) no differences were observed at follow-up.

## Introduction

Dementia, including Alzheimer's disease, is expected to affect to 75.62 million people by 2030 and 135.46 million by 2050. The prevention and treatment of secondary causes of dementia (hypothyroidism, vitamin B12 deficiency, Lyme disease, neurosyphillis…) and the control of risk factors (smoking, underactivity, obesity, hypertension, diabetes, lack of education…) may decrease the incidence (Prince et al., 2013). Research focused on finding a cure for dementia is key, but in the meantime, we must bear in mind that patients with dementia need the most appropriate treatment. New drugs and non-pharmacological treatments are currently being researched.

Animal-assisted therapy (AAT), the use of animals in therapy sessions, is one such non-pharmacological tool currently under investigation. The Cochrane Database of Systematic Reviews published a protocol to study “AAT for people with serious mental illness” (Downes et al., 2013). In 2006, the British National Institute for Health and Clinical Excellence (NICE) published guidelines for people with dementia (Fairbairn, 2006) and included AAT as an approach that may be considered a non-pharmacological intervention for non-cognitive symptoms and behavior. AAT seems to calm agitated behavior and has positive effects on the quality of social interaction and mood disturbances, although no effect was observed on cognitive performance (Bernabei et al., 2013). The presence of an animal involves an increase in the frequency and duration of visual contact and smiles. Interaction with a real animal, rather than a stuffed one, increases the frequency of verbalization. Studies have shown a decrease in verbal aggression and agitation with reduced behavior problems, although the need for drug treatment was not changed. However, it has been observed that agitation increases when therapy with animals was withdrawn. The effect of animal therapy was independent of the severity of the dementia (Filan and Llewellyn-Jones, 2006).

However, AAT is not always possible. Animals are often not allowed in nursing homes or day care centers, due to the risk of injury to patients, staff or visitors, the possibility of allergic reactions, and the potential nuisance of cleaning up after the animals. Patients or staff may have undesired reactions to animals, both negative (i.e., Fear) and overly positive (i.e., becoming too attached). Aggressive patients could frighten or harm the animals. And the cost of animal care (space, time and money) might exceed the benefits of a few hours of therapy per day.

Thus, the alternative of replacing real animals with animal-shaped objects became an object of investigation (Nakajima et al., 2001). In recent years, social robots have been also used as reasonable substitutes for animals in therapy for people suffering from dementia (Wada et al., 2008; Shibata, 2012).

Robots have less needs for space, time, or care. Their sensors can respond to environmental changes (movements, sounds…) simulating interaction with the patient. They can monitor patients or be used in the therapy. Other potential benefits of therapy with robots are that there are no known adverse effects, specially trained personnel are not required and they can repeat the script in the same way as many times as it is required.

In 2009, a systematic review examining the literature on the effects of assistive social robots in health care for the elderly, especially in the role of providing companions for patients, was published (Broekens et al., 2009). The main conclusions were that most of the elderly people liked the robots and the robots can improve: health (by lowering levels of stress and increasing immune system response), mood (decreasing feelings of loneliness) and communication (increasing it). Moreover, the robots lessened the severity of dementia as measured by specific scales in some studies.

Bemelmans et al reviewed the literature in 2012 and found that the most of the studies reported positive effects of companion-type robots on (socio) psychological (e.g., mood, loneliness, and social connections and communication) and physiological (e.g., stress reduction) parameters (Bemelmans et al., 2012).

In the present study, animals and robots were added into the therapy sessions at a center for dementia patients. They were employed just as any other tool the therapists might use, in order to discover the potential effect of the tool without changes in the therapists' actions, the session content or the environment of the patients.

## Objective

Pilot studies were carried out in order to test the effect of introducing a humanoid robot (NAO), a pet robot (PARO) and a real trained animal (DOG) in the therapeutic sessions for patients with dementia in relation to behavior changes, apathy and quality of life.

In a nursing home, where institutionalized patients with dementia are living in controlled conditions, the objective was to compare the effects of therapy sessions involving:

- (Phase 1) a humanoid robot (NAO), who is able to use oral language (phrases previously recorded) and move like a human; an animal-shaped robot (PARO), who does not use oral language but sounds and moves like an animal; and with conventional therapy (CONTROL).
- (Phase 2) a trained dog (DOG); an animal-shaped robot (PARO), who has been used as reasonable substitute for animals in therapy for people suffering from dementia; and with conventional therapy (CONTROL).

In a day care center, where dementia patients attend for 8 h a day approximately, the objective was to compare the baseline with the follow-up effects of therapy sessions involving the robots:

- (Phase 1) NAO and
- (Phase 2) PARO.

## Materials and methods

### Patients

All dementia patients being cared for at the Alzheimer Center Reina Sofía Foundation (ACRSF) (Olazarán et al., 2012), a public nursing home and day care center, were invited to participate. All the participants, and their legal guardians or families, received information and signed an informed consent form approved by the CIEN Foundation Ethical Committee. Inclusion criteria were a diagnosis of neurodegenerative dementia, being cared for at ACRSF and possessing a signed consent form. Exclusion criteria were: fear of the robot or dog and severe acute illness (requiring hospitalization or intensive medical care).

### Therapeutic tools

**The robots used in this study were:**

#### PARO

A social robot with the appearance, movement and sounds of a baby seal. It has programmable behavior and sensors for posture, touch, sound, and light. Its eyes, which are big, black and with long eyelashes, can open and close; it can also move its neck (laterally and up-and-down), anterior flippers and tail. Although its movements are silent, it emits short and sharp squeals like a real seal. It is very soft and white in color, with hard Velcro covering the access to the mechanism (so it is not easy to access it during therapy sessions). It cannot move forward or change its sounds and weighs 2.7 kg.

#### NAO

A white humanoid robot, measuring 58 cm tall and weighing 4.3 kg. It has sensors for movement, touch, sonar, sound, and vision. It can talk and sing. It has a robotic voice, but it is possible to replace it with mp3 recordings of a child-like human voice that is easier for patients to understand. It can move its neck and arms, walk, or dance. Software was developed to allow the robot to act out a script for therapy sessions. These scripts included effects like speech, music and movements. During the therapy session, the therapist could control the activation of and progression through the script using remote control software installed in an Android device. The therapists were able to pause the script, repeat sections of it, or jump to another section. It was also possible to use this software to remotely operate the robot in order to make it walk or move its head (Martin et al., 2013).

**The animals used were dogs:** two adult black Labrador Retrievers. Both had received prior training for therapy. Each dog participated in half of the sessions with each group. The therapists received training prior to the sessions on the use of animals, and the animals were allowed to adapt to the Center before beginning the research activities. Therapists specially trained to work with animals attended all the sessions with the dogs, in order to monitor the course of therapy.

**Otherwise, the tools used in the control group were the same as in the other two groups**. It was necessary to adapt certain tools used in the sessions for its use with the robots and the dogs: specially designed vests with pockets and Velcro were produced, and flash cards were laminated.

### Design

#### Nursing home

A controlled clinical trial of parallel groups, randomized by blocks (living units) and stratified by dementia severity, comparing therapy with robots and dogs against standard care was carried out.

One hundred fifty six patients with dementia reside in the ACRSF nursing home. All patients receive similar care, in terms of: medical and custodial care, non-pharmacological therapy and personalized nutrition, therapy programming, and physical exercise. They live in similarly designed floors, with natural lighting and large spaces tailored to their needs. Residents live on different floors or living units depending on the severity of their dementia. The floors with patients of similar severity were grouped by threes: three floors of patients with mild-moderate dementia, three floors of patients with moderate-severe dementia, and three floors of patients with severe dementia. For each dementia severity group, each floor was randomly assigned to one of the three therapies (randomization by blocks). All the environmental conditions were controlled for, so that the specific tool used by the therapist in the sessions was the only difference in the sessions experienced by the different therapy groups.

The study had 2 year-long phases, carried out during two consecutive years (2012 and 2013), comparing two different modalities of experimental therapies to each other and to standard care. The therapies compared were:

- Phase 1: CONTROL, NAO (humanoid social robot), PARO (animal-esque social robot).
- Phase 2: CONTROL, PARO (animal-esque robot), DOG (real animal).

All the patients included were assigned to only one of the three therapeutic groups, worked with only one tool (Control, PARO, NAO, or DOG) and were evaluated before and after the study sessions. Randomization was performed before the baseline evaluations using a six-sided die.

#### Day care center

Forty people are cared for at the ACRSF day care center. A pretest-posttest design was used, due to the small number of participants and the inability to control the differences between their medical and nursing care, routines and nutrition. All patients participated in sessions with only one of the therapeutic tools: NAO in the first phase and PARO in the second. Patients were divided by the therapists in two therapeutic groups according to dementia severity: mild-moderate dementia and moderate-severe dementia.

### Therapy

The therapy sessions were performed 2 days a week during 3 months.

All therapeutic sessions were conducted by the same therapist, with the same structure as the other therapeutic programs, at the same time of day and for the same duration of time (30–40 min).

The therapists were certified occupational and physical therapists, and neuropsychologists employed by the ACRSF. They received instructions on the implementation and possible uses of robots and animals as they had no previous expertise in this area. The animal therapists and robot engineers did not participate in the therapy; they only monitored the session from one side of the room, out of the patients' view. Session guides were written and followed in every session.

The therapists used the same model of standard therapy, introducing the experimental tools as one more element of the therapy. It is important to note that the object of investigation was the effect of the specific tools, not the effect of the therapy itself, so the tools had to be used for the same purpose and in the same way in the three therapeutic groups if possible. Only one robot or dog was used in every session.

The patient interacted with the robots, the animals and the therapists to perform several therapeutic activities, including: identifying numbers, words, and colors using flash cards; practicing the use of everyday objects such as combs; sensory stimulation exercises using different textured fabrics…

The robots and the animals were wearing specially designed vests with pockets and Velcro, in order to carry the objects used in the sessions, and move from patient to patient.

All sessions had the same overall structure: greeting the group, introduction, therapeutic exercises (cognitive or physical therapy) and ending.

The introduction included the presentation of the target tool, orientation activities (spatial, temporal, and personal orientation), and motivation to participate in the therapy session.

Therapeutic exercises were small units of activities, focused on the stimulation of memory, language, calculation, movement, praxis, and the use of the different senses. Activities involved physical exercises, questions and answers, music, videos, and manipulation or touching several objects. Between the exercises, there were brief pauses to encourage the collaboration and participation of all users.

At the end of the session, the therapists reviewed what the group did with everyone, asked whether or not they liked participating in the sessions, and lead the group in a farewell song.

Group sessions were employed for patients with mild or mild-moderate dementia, and individual sessions were used with patients with moderate-severe and severe dementia. The group sessions were conducted with 9–15 participants seated in a circle with the therapist and the tools in the inside, moving from patient to patient. In the individual sessions, the therapist was sitting in front of the patient, at the same level, providing stimuli one by one.

Sessions with four levels of difficulty were designed:

1. Mild difficulty level (performed with patients from the day care center). An extensive cognitive session was designed, and the therapist selected a different part of the session each day to avoid repetitiveness and the participants' boredom. The physical therapy session consisted of a complete set of exercises involving head, neck and upper and lower limbs. The robot was programmed to move faster, given the better physical condition of the majority of the patients from the day care center.
2. Mild-moderate level of difficulty. Three sessions were designed: therapy with music, cognitive therapy and physical therapy.
3. Moderate-severe difficulty level. Two sessions were designed: cognitive therapy and physical therapy.
4. Severe difficulty level. One session was designed using language stimulation, music, passive movement and sensory stimuli.

All therapy sessions were recorded on video for *post-hoc* observational analysis. Two cameras with tripods were used and were placed outside the circle formed by the patients. There were several recording sessions previous to the start of the study to get the patients and therapists used to its presence, thereby reducing the Hawthorne effect.

### Assessments

Evaluation was carried out by blinded raters at baseline and follow-up using the following validated scales:

- The Global Deterioration Scale (GDS) (Reisberg et al., 1982), which was given by a neurologist,
- The Severe Mini Mental State Examination (sMMSE) (Harrell et al., 2000; Buiza et al., 2011) and the Mini Mental State Examination (MMSE) (Folstein et al., 1975; Lobo et al., 1999), which were given by a neuropsychologist,
- The Neuropsychiatric Inventory (NPI) (Cummings et al., 1994; Vilalta-Franch et al., 1999; Boada et al., 2005), the Apathy Scale for Institutionalized Patients with Dementia Nursing Home version (APADEM-NH) (Agüera-Ortiz et al., 2015), which was used with patients in the nursing home only, and the Apathy Inventory (AI) (Robert et al., 2002), which was used with patients from the day care center only, and was given by a psychiatrist,
- And the Quality of Life in Late-stage Dementia (QUALID) (Weiner et al., 2000; Garre-Olmo et al., 2010), which was given by a sociologist.

When the evaluations required interviewing the nursing staff about patient functioning, the raters interviewed the same staff members for each patient at baseline and follow up whenever possible.

Medical information was also collected for subsequent analysis. The wash-out period between phases was 9 months long.

### Data analysis

Statistical analysis, apart from descriptive statistics, included the Wilcoxon, Mann-Whitney and Kruskal-Wallis tests for comparisons, performed by a blinded researcher. Non-parametric tests were used as the data did not meet the assumptions for use of parametric statistics. The statistical analysis was done using Stata software (Stata^©^. Stata Corp., College Station, Texas, USA version: 15).

## Results

All patients, families and legal guardians received written information and informational meetings were organized.

One hundred and forty eight people signed the informed consent forms. Before the first evaluation, 22 participants died, two people moved to another center and one person withdrew consent.

In the first experimental phase, two people suffered acute illnesses and did not complete the treatment. During the wash out period, 31 additional people signed the consent forms, 12 patients died, nine people moved to other centers and one person withdrew consent.

**Figure 1:**
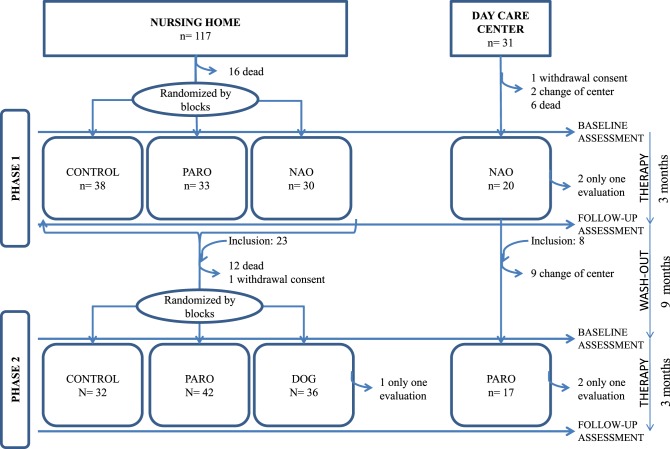
**All patients whose families/guardians gave consent and who fit inclusion/exclusion criteria were included**. Therapy sessions: each group worked only with a tool (conventional, PARO, NAO, or DOG) in the nursing home; in the day care center all participants worked with NAO in the first phase, and PARO in the second. Assessments were realized before and after the study sessions. The wash-out period allowed the entry of new participants. Randomization was carried out before the study sessions only in the nursing home.

In the second experimental phase, three people did not complete the treatment due to illnesses or absences from ACRSF (Figure 1).

### Nursing home

#### Phase 1

In the first phase, 101 patients with moderate/severe dementia (GDS 4: 2%, GDS 5: 17%, GDS 6: 44% and GDS 7:37%), mean age 84.68 years old (range: 58–100 years), 88% of which were women, were included.

Dementia diagnosis was: 84.2% Alzheimer disease, 10.9% mixed dementia, 3% Parkinson's disease dementia, 1% dementia with Lewy bodies, 1% Frontotemporal dementia.

There were 38 people in the CONTROL group, 33 in the PARO group and 30 in the NAO group. Evaluation showed no significant differences between groups at baseline.

All groups showed a statistically significant increase in GDS scores, indicating a decreased functional level (Fisher's exact < 0.000) at follow-up.

**Figure 2:**
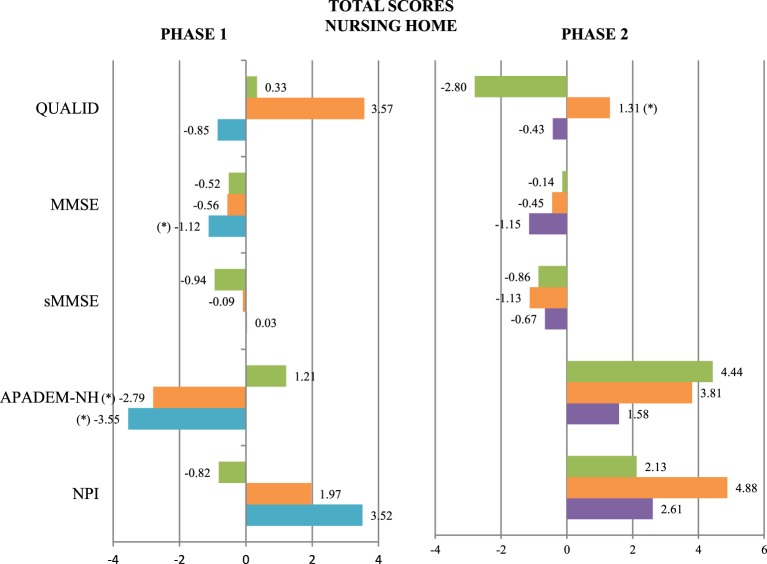
**Mean change at follow-up evaluation in the QUALID, MMSE, sMMSE, APADEM-NH, and NPI scores in the three patients groups of Phase 1 and Phase 2**. CONTROL (green), PARO (orange), NAO (blue), and DOG (purple) (^*^): Statistically significant differences between the groups at follow-up were observed. *P* not corrected < 0.05. The scales are represented on the vertical axis and the mean change in the units of measure of each scale is represented on the horizontal axis.

Scores on QUALID, sMMSE, and NPI (total score) showed no statistically significant changes between groups at follow-up (Figure 2).

**Figure 3:**
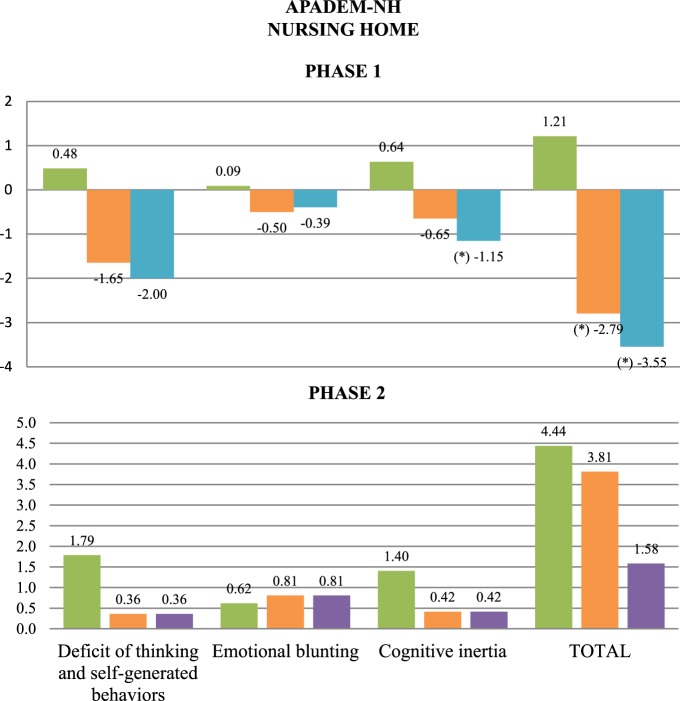
**Mean change at follow-up in APADEM-NH scores, by item and total, for the three patients groups of Phase 1 and Phase 2**. CONTROL (green), PARO (orange), NAO (blue), and DOG (purple) (^*^): Statistically significant differences between the groups at follow-up were observed. *P* not corrected < 0.05. The scales are represented on the horizontal axis and the mean change in the units of measure of each scale is represented on the vertical axis.

**Figure 4:**
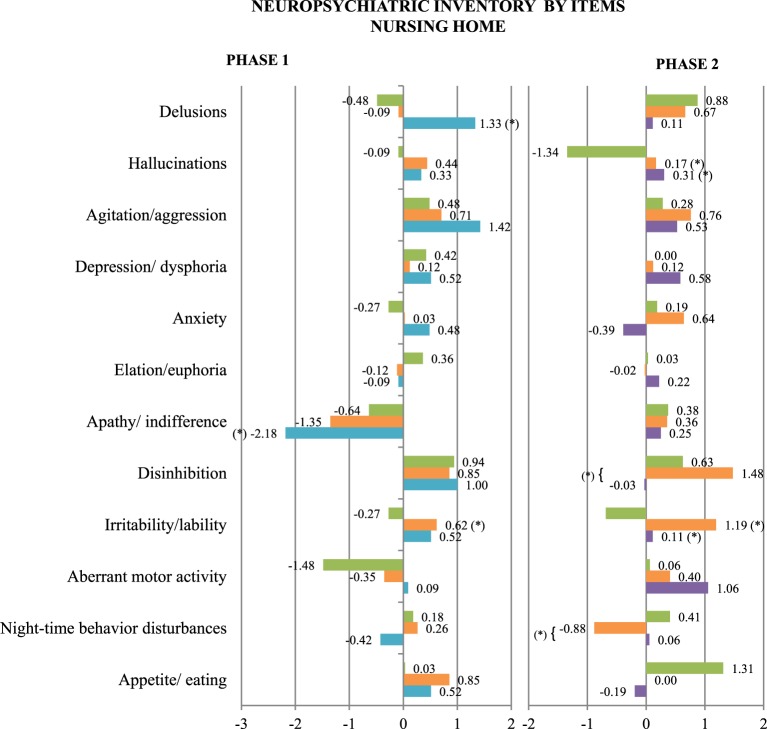
**Mean change at follow-up in NPI scores, by item, for the three patients groups of Phase 1 and Phase 2**. CONTROL (green), PARO (orange), NAO (blue), and DOG (purple) (^*^): Statistically significant differences between the groups at follow-up were observed. *P* not corrected < 0.05. The scales are represented on the vertical axis and the mean change in the units of measure of each scale is represented on the horizontal axis.

In contrast, statistically significant differences were found after treatment in MMSE scores (a significant decrease in the NAO group), APADEM-NH scores (both the PARO and the NAO groups had significant decreases in total score, and the NAO group in cognitive inertia score), and several NPI items: delusions (a significant increase in the NAO group), apathy (a significant decrease in the NAO group) and irritability/lability (a significant increase in the PARO group) (Figures 3, 4 and Table 1).

**Table 1:** **Baseline and follow-up means, standard deviation (SD) and *p*-value (P) in every variable of the study with statistically significant change in the patient groups [CONTROL (C), PARO (P), NAO (N), and DOG (D)] in the nursing home and the day care center**.

			**Variable**	**CONTROL**				**PARO**				**P C-P**	**NAO**				**P C-N**	**P P-N**
				**Baseline mean**	***SD***	**Follow-up mean**	***SD***	**Baseline mean**	***SD***	**Follow-up mean**	***SD***		**Baseline mean**	***SD***	**Follow-up mean**	***SD***		
Nursing home	Phase 1	MMSE		3.64	5.42	3.12	4.72	3.20	4.95	2.74	4.72	0.282	3.55	5.18	2.42	4.49	**0.022**	0.145
		SMMSE		8.67	10.14	7.73	9.56	7.97	8.84	8.12	9.68	0.239	7.76	8.65	7.79	9.00	0.702	0.586
		APADEM-NH	Total	43.21	21.80	44.42	23.59	48.40	19.12	44.74	22.29	**0.049**	45.06	20.69	41.52	22.59	**0.030**	0.564
			Cognitive inertia	10.73	5.89	11.36	6.32	11.97	5.27	11.15	5.79	0.251	12.03	5.48	10.88	6.30	**0.034**	0.313
		NPI	TOTAL	27.36	14.01	26.55	16.05	22.17	12.96	24.44	17.10	0.246	27.94	15.03	31.45	21.55	0.238	0.782
			Delusions	0.91	1.99	0.42	1.54	0.66	1.98	0.59	2.23	0.455	0.79	1.82	2.12	3.90	**0.011**	0.063
			Apathy/indifference	8.73	2.54	8.09	3.43	9.26	2.28	7.82	3.35	0.292	8.85	2.55	6.67	3.96	**0.047**	0.275
			Irritability/lability	2.58	2.97	2.30	3.02	1.89	2.99	2.56	3.78	**0.033**	3.21	3.66	3.73	3.67	0.085	0.728
			**Variable**	**CONTROL**				**PARO**				**P C-P**	**DOG**				**P C-D**	**P P-D**
				**Baseline mean**	***SD***	**Follow-up mean**	***SD***	**Baseline mean**	***SD***	**Follow-up mean**	***SD***		**Baseline mean**	***SD***	**Follow-up mean**	***SD***		
	Phase 2	QUALID		27.70	7.82	24.72	6.68	25.44	7.56	26.75	8.16	**0.044**	24.91	7.39	24.33	6.68	0.101	0.547
		NPI	Total	26.53	20.33	28.66	19.08	24.40	16.15	29.29	19.60	0.294	19.72	15.15	22.33	14.67	0.649	0.364
			Hallucinations	1.81	3.18	0.47	1.54	0.93	2.32	1.10	1.92	**0.020**	0.17	0.74	0.47	1.21	**0.004**	0.625
			Disinhibition	0.91	2.68	1.53	3.36	0.62	1.41	2.10	3.88	0.172	0.92	2.52	0.89	2.61	0.599	**0.026**
			Irritability/lability^*^	3.78	3.31	3.09	4.17	2.14	3.06	3.33	4.23	**0.003**	2.14	3.29	2.25	2.93	**0.024**	0.873
			Night-time behavior disturbances	1.16	2.26	1.56	3.19	2.43	3.51	1.55	2.78	0.078	1.11	2.69	1.17	2.36	0.771	**0.028**
Day care C.	Phase 1		**Variable**	**NAO**				**P**										
				**Baseline mean**	***SD***	**Follow-up mean**	***SD***											
		NPI	TOTAL	33.65	21.91	22.47	12.89	**0.007**										
			Irritability/lability	3.25	3.41	1.57	2.67	**0.046**										

* *Significant differences between groups at baseline evaluation. P not corrected < 0.05. Bold values are p < 0.05*.

There were no statistically significant differences between the NAO and PARO groups.

#### Phase 2

In the second phase, 110 patients with moderate/ severe dementia (GDS 5: 22%, GDS 6: 30%, and GDS 7: 48%), mean age 84.7 years old (range: 59–101 years), 90% of which were women, were included.

Dementia diagnosis was: 88.2% Alzheimer disease, 7.3% mixed dementia, 3.6% Parkinson's disease dementia, 1.8% dementia with Lewy bodies, 0.9% Frontotemporal dementia.

There were 32 people in the CONTROL group, 42 in the PARO group and 36 in the DOG group. Evaluation showed no significant differences between groups at baseline, except on the NPI irritability item (CONTROL group: 3.78 ± 3.3; PARO group: 2.14 ± 3.05; DOG group: 2.13 ± 3.28; *p* = 0.0215).

All groups showed a statistically significant increase in GDS scores, indicating a decreased functional level (Fisher's exact < 0.000) at follow-up.

There were no statistically significant differences in MMSE, sMMSE, APADEM-NH, and Total NPI scores between groups at follow-up (Figure 2).

On the contrary, statistically significant differences were found after treatment in QUALID scores (a significant increase in the PARO group), and several NPI items: hallucinations and irritability/lability (a significant increase in the PARO and the DOG groups vs. the CONTROL group than decreases), disinhibition (a significant increase in the PARO group vs. the DOG group) and night-time behavior disturbances (a significant decrease in the PARO group vs. the DOG group) (Figure 4 and Table 1).

### Day care center

#### Phase 1

In phase 1, 20 patients with moderate/severe dementia, mean age 77.9 years (range: 68–87), 50% women, were included.

Dementia diagnosis was: 75% Alzheimer disease, 15% mixed dementia, 5% Parkinson's disease dementia, 5% dementia with Lewy bodies.

After the sessions with NAO, follow-up evaluation showed: an increase in GDS scores (changes from baseline to follow-up: GDS 3:15–10%; GDS 4: 5% (no change); GDS 5: 40–30%; GDS 6: 25–30%; GDS 7: 15–25%). There were no statistically significant changes in sMMSE and MMSE scores.

**Figure 5:**
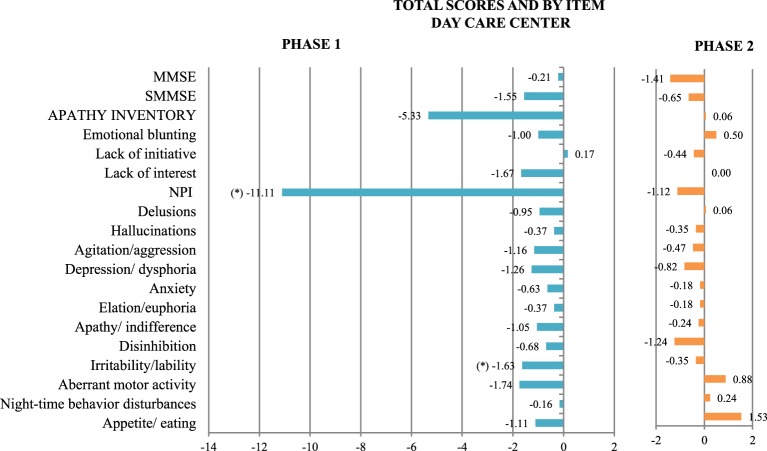
**Mean change at follow-up in scores on all the scales used, both totals and by item, in the day care center group of Phase 1, with NAO (blue), and Phase 2, with PARO (orange)**. (^*^) Statistically significant differences between the groups at follow-up were observed. *P* not corrected < 0.05. The scales are represented on the vertical axis and the mean change in the units of measure of each scale is represented on the horizontal axis.

Significant decrease was seen in: NPI-irritability/lability scores and total NPI scores (Figure 5 and Table 1).

#### Phase 2

In the second phase, 17 patients with moderate/ severe dementia, mean age 79 years (range: 69–87), 58.8% women, were included.

Dementia diagnosis was: 82.4% Alzheimer disease and 17.6% mixed dementia.

At follow-up, after sessions with PARO: GDS scores increased (GDS 4:5.88–0%; GDS 5: 41.18% (no change); GDS 6: 35.29–29.4%; GDS 7: 17.65–29.4%). There were no statistically significant changes in sMMSE and MMSE scores, or in any other variable (Figure 5).

## Discussion

To our knowledge, this is the first study, in one single center, where an animal-esque robot, a humanoid robot and trained therapy dogs were compared as potential tools for therapy for people with dementia. These new tools were the only change introduced to the patients by the intervention, because changes in personnel and environment were carefully controlled for limiting the effect of possible confounding effects.

In order to control for environmental factors, the regular therapists performed the sessions, rather than new therapists trained in the use of robots or dogs who would be unfamiliar to the patients. However, while there are therapists who specialize in animal therapy, there are currently no therapists who specialize in therapy using robots. Additionally, while the application of animal-esque robots in therapy targeted toward people with dementia has been documented, to our knowledge this is the first time that the NAO robot has been used for this purpose. Therefore, although some more training in AAT might have improved the results, it might have also introduced a bias in favor of the dog, because there is greater body of knowledge and more training materials concerning AAT than robot assisted therapy.

The measures used were internationally validated scales which have proven to be sensitive and specific measures of the target symptoms. All evaluations were performed by professionals trained in the use of the measures. The changes observed after the introduction of these new tools affected all of the investigated variables:

**Quality of life (QoL)**, measured with the QUALID scale, was only studied in the nursing home groups. In the first phase no changes were observed, but in the second one, the group of patients who worked with the conventional therapy showed improvement, while the group who worked with the animal robot slightly worsened. In 2009 Tapus et al. proposed a customized protocol for the use of social auxiliary robots as tools to improve the QoL of people with dementia, through motivation, encouragement, and companionship for users suffering from cognitive changes related to aging and/or Alzheimer's disease (Tapus, 2009). A social robot, AIBO, was used for 7 weeks with community-residing and institutionalized elderly and incapacitated patients. These patients showed significant improvements in QoL as measured by some health-related QoL questionnaires (Kanamori et al., 2003). In contrast, our study has barely shown significant changes in QoL, with the only change being a slight decrease in the QoL of patients in the animal robot group.

**Global deterioration scale** and **cognitive state** (measured with MMSE and sMMSE) worsened in all the follow-up evaluations as was expected in a progressive and degenerative disease. Only one exception was observed: after the use of NAO, in the nursing home, the sMMSE remained the same. In the same group and experiment, a significant decrease in the MMSE score was observed. Although the MMSE and the sMMSE are both screening scales for cognitive decline, the sMMSE is more appropriate for people with moderate and severe dementia, such as the people who participated in this program. A literature review found that animal-assisted interventions with elderly patients with dementia has a positive effect on communication and coping ability, but not on cognitive performance (Bernabei et al., 2013). In 2008, an improvement in dementia patients' cortical neuronal activity was observed using a 21-channel EEG after the use of PARO (Wada et al., 2008). Several studies (Tapus et al., 2009; Chan and Nejat, 2010; Fasola and Mataric, 2010) describe the use of social robots as a tool for monitoring and encouraging cognitive activities of the elderly and/or individuals suffering from dementia as improving task performance and reducing user frustration. Increased cortical neuronal activity or greater motivation to perform and complete cognitive tasks could lead to a better cognitive test outcome in social robots groups, but in this study, changes between groups were not observed.

Twelve **neuropsychiatric symptoms** were analyzed via the NPI, the APADEM-NH and the AI. **Apathy** is a prominent symptom in dementia. The APADEM-NH scale, a tool developed at the ACRSF, was used to measure apathy. The scale has the advantage of accurately measuring apathy independently of the patient's degree of dementia or depression. As the APADEM-NH is not suitable for people who are not living in a nursing home, the AI was used in the day care center. The apathy item of the NPI scale was additionally used to examine apathy. In the nursing home, in the first phase, statistically significant improvement was seen in the total scores on the APADEM-NH of patients in both robot groups, and in the scores on the apathy item of the NPI scale and the cognitive inertia item of the APADEM-NH of patients in the NAO group. This results replicate previous studies were, despite a lack of methodological rigor, it is apparent that non-pharmacological interventions have the potential to reduce apathy in dementia (Brodaty and Burns, 2012). In the second phase, no statistically significant difference in measures of apathy was found. An explanation could be that apathy in institutionalized patients with advanced dementia seems to increase in time (Wetzels et al., 2010) and therapeutic interventions may have a window in mild and moderate dementia, but not in advanced dementia (López and Agüera-Ortiz, 2014). In the day care center, there was no statistically significant difference in apathy.

Analysis of the NPI scores of all patients in this study reveals a random assortment of changes that were minimal at best. Statistically significant changes were observed in:

- The NAO group:
  ◦ Impairment in delusions and
  ◦ Improving in apathy (Phase 1 nursing home), total score and irritability/lability (day care center)
- The PARO group:
  ◦ Impairment in irritability/lability (nursing home), hallucinations (Phase 2 nursing home) and disinhibition (vs. the DOG group)
  ◦ Improving in night-time behavior disturbances (vs. the DOG group).
- The DOG group:
  ◦ Impairment in hallucinations, irritability/lability

Measures of irritability on the NPI item in the nursing home groups at baseline for Phase 2 showed statistically significant differences between groups, which casts some doubt on the significance of the follow up results.

The changes observed in hallucinations and in the DOG group were produced more by the improvement in the other therapeutic groups, than by its impairment.

A decrease in agitation after the use of animal assisted interventions has been described in the literature (Churchill et al., 1999; Richeson, 2003; O'Neil et al., 2011; Bernabei et al., 2013), as a decrease in behavioral symptoms after the use of a therapy dog during the day time hours (*p* < 0.05) with no significant differences during the evening hours (McCabe et al., 2002). Behavioral improvement, as defined by a reduction in anxiety and aggressiveness, has also been previously reported after the use of PARO (Shibata and Coughlin, 2014). These previous findings were not replicated in this study.

## Limitations

The study was designed in order to minimize any potential biases: 3 months of therapy were used in order to decrease the novelty effect, the raters and statistician were blinded, several recording sessions were held before the study began to get the patients and therapists used to the presence of the camera and decrease the Hawthorne effect, interaction between ACRSF regular caregivers and the robots and dogs was avoided to reduce potential informant bias, and while active participation in the therapy sessions was encouraged in order to decrease selection bias patients were not forced to interact with the therapist or the tools.

Throughout the study, several participants left the center or unit or died, and several patients joined the study late. Most of these changes did not occur during the interventions. Some participants were only evaluated once, due to hospitalization, illness or absence from the center: only 1.6% of participants were lost to follow up in the first year and 2.3% in the second.

This study was a pilot study in a sample, not representative of the general population, with a low number of participants. The changes after the use of the three new tools were minimal, and some of the statistical significant changes may be false positives, due to the high number of comparisons made and to the relatively small sample sizes. It is not possible to conclusively state that these tools were the cause of the changes seen, because of several possible confounding factors (i.e., differences in participants' pharmacologic regimens or type of dementia). However, these factors were controlled for, and will be investigated in future research.

In the nursing home, randomization was done by unit, not by individual participant. Although individual randomization would have been optimal, it would have required moving residents to different units for the sessions and would have introduced major changes in the daily routines and environments of the participants. Additionally, the environments and the characteristics of the individuals in units of the same dementia level are very similar. Baseline evaluation showed no difference between any of the nursing home groups on any of the measures (except in irritability in the nursing home in Phase 2), indicating that there is no significant difference between people who live in different units in the variables analyzed in this study.

## Concluding remarks

In a controlled pilot study of parallel groups in institutionalized patients with moderate/severe dementia comparing 3 months of assisted therapy with:

- A humanoid robot, an animal-shaped robot and conventional therapy, the main findings were: a decrease in apathy in the humanoid and animal shaped robot groups; increased delusions in the group treated with the humanoid robot and increased irritability in both robot groups; and a decrease in scores on the MMSE, but not the sMMSE, in the humanoid robot group. There were no statistically significant differences between the humanoid and animal shaped robot groups.
- An animal-shaped robot, a real therapy dog and conventional therapy, the main findings were: a decrease in quality of life in the animal shaped robot group compared to the conventional therapy group; increased hallucinations and irritability in both the robot and animal groups compared to the control group; increased disinhibition in the animal-shaped robot group and decreased disinhibition in the humanoid robot group, decreased night-time behavior disturbances in the animal-shaped robot group and increased night-time behavior disturbances in the dog group.

In a study of robot therapy sessions for patients with moderate/severe dementia cared for at a day care center, participants showed improvements in irritability and global neuropsychiatric symptoms after participating in sessions with the humanoid robot, but not after sessions with the animal-shaped robot.

## Future studies

Randomized controlled trials are needed with a larger amount of patients, in order to better understand the effects of robots and dogs on the therapy of people with dementia.

Additionally, new scales that are internationally validated and more sensitive and specific are needed, in order to detect the slight changes in behavior, emotion and cognition that were observed during the session but were not significant enough to appear in the analysis.

As a result of this study, our team is going to focus on the use of humanoid robots in cognitive therapy for people with mild dementia and in the use of pet robots for people with moderate to severe dementia.

## Grants

This study received grants from the Spanish Ministry of Science and Innovation (PI10/02567) and the Spanish Ministry of Health, Social Policy and Equality (IMSERSO 231/2011).

### Conflict of interest statement

The authors declare that the research was conducted in the absence of any commercial or financial relationships that could be construed as a potential conflict of interest.
